# Fine particulate matter aggravates smoking induced lung injury via NLRP3/caspase-1 pathway in COPD

**DOI:** 10.1186/s12950-024-00384-z

**Published:** 2024-04-24

**Authors:** Chiwook Chung, Suk Young Park, Jin-Young Huh, Na Hyun Kim, ChangHo Shon, Eun Yi Oh, Young-Jun Park, Seon-Jin Lee, Hwan-Cheol Kim, Sei Won Lee

**Affiliations:** 1 Department of Pulmonary and Critical Care Medicine, Asan Medical Center, University of Ulsan College of Medicine, 88 Olympic-ro 43-gil, Songpa-gu, 05505 Seoul, Republic of Korea; 2 Department of Pulmonary and Critical Care Medicine, Gangneung Asan Hospital, University of Ulsan College of Medicine, Gangneung, Republic of Korea; 3 Division of Pulmonary, Allergy and Critical Care Medicine, Department of Internal Medicine, Chung- Ang University Gwangmyeong Hospital, Chung-Ang University College of Medicine, Gwangmyeong, Republic of Korea; 4 Efficacy Evaluation Center, WOOJUNGBIO Inc, Hwaseong, Republic of Korea; 5 Department of Physiology, Yonsei University College of Medicine, Seoul, Republic of Korea; 6 Korea Research Institute of Bioscience and Biotechnology, Daejeon, Republic of Korea; 7 Department of Occupational and Environmental Medicine, College of Medicine, Inha University, Incheon, Republic of Korea

**Keywords:** PM_2.5_, Smoking, Pyroptosis, NLRP3, COPD

## Abstract

**Background:**

Exposure to noxious particles, including cigarette smoke and fine particulate matter (PM_2.5_), is a risk factor for chronic obstructive pulmonary disease (COPD) and promotes inflammation and cell death in the lungs. We investigated the combined effects of cigarette smoking and PM_2.5_ exposure in patients with COPD, mice, and human bronchial epithelial cells.

**Methods:**

The relationship between PM_2.5_ exposure and clinical parameters was investigated in patients with COPD based on smoking status. Alveolar destruction, inflammatory cell infiltration, and pro-inflammatory cytokines were monitored in the smoking-exposed emphysema mouse model. To investigate the mechanisms, cell viability and death and pyroptosis-related changes in BEAS-2B cells were assessed following the exposure to cigarette smoke extract (CSE) and PM_2.5_.

**Results:**

High levels of ambient PM_2.5_ were more strongly associated with high Saint George’s respiratory questionnaire specific for COPD (SGRQ-C) scores in currently smoking patients with COPD. Combined exposure to cigarette smoke and PM_2.5_ increased mean linear intercept and TUNEL-positive cells in lung tissue, which was associated with increased inflammatory cell infiltration and inflammatory cytokine release in mice. Exposure to a combination of CSE and PM_2.5_ reduced cell viability and upregulated NLRP3, caspase-1, IL-1β, and IL-18 transcription in BEAS-2B cells. NLRP3 silencing with siRNA reduced pyroptosis and restored cell viability.

**Conclusions:**

PM_2.5_ aggravates smoking-induced airway inflammation and cell death via pyroptosis. Clinically, PM_2.5_ deteriorates quality of life and may worsen prognosis in currently smoking patients with COPD.

**Graphical Abstract:**

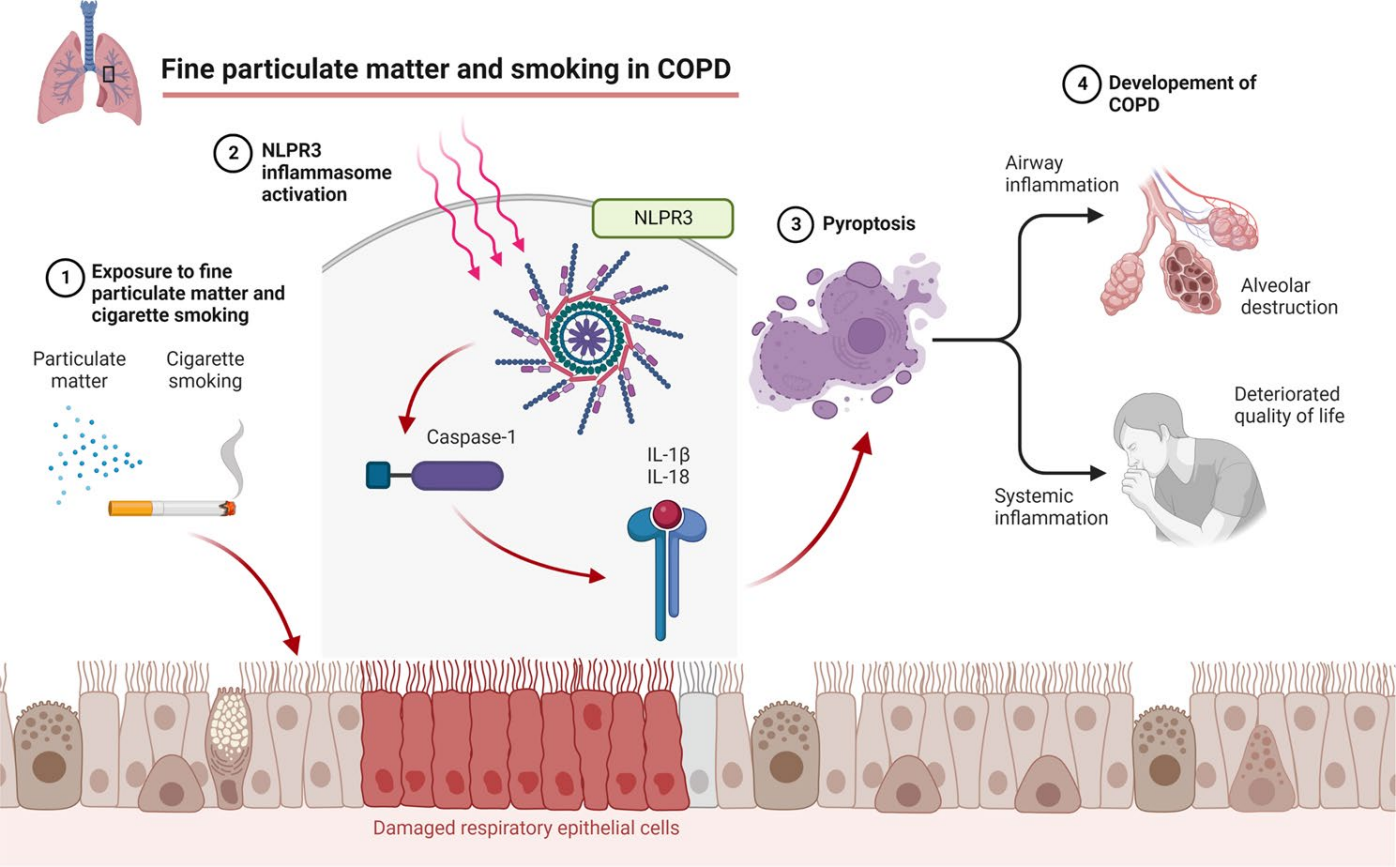

**Supplementary Information:**

The online version contains supplementary material available at 10.1186/s12950-024-00384-z.

## Background

Chronic obstructive pulmonary disease (COPD) is a chronic respiratory disease usually caused by prolonged exposure to noxious gases or particles [1]. Although cigarette smoking is an important risk factor in COPD, many patients with COPD are never-smokers [2–4]. Occupational exposure and biomass fuels are well-known risk factors in never-smoker COPD [5, 6]. Recent studies have linked particulate matter of diameter ≤ 2.5 μm (PM_2.5_) to decreased lung function, airway inflammation, and emphysematous changes in the lungs, leading to the development of COPD and increased mortality [7–10].

Both PM_2.5_ and smoking have been reported to promote inflammation and cell death in the lungs [11–16]. Particularly, PM_2.5_ is known to induce various types of cell deaths, including autophagy, necrosis, apoptosis, pyroptosis, and ferroptosis [17]. Recently, pyroptosis has been identified as a crucial process in lung injury. Pyroptosis is an inflammatory type of programmed cell death mediated by caspase-1 and activated by the inflammasome [18, 19]. The inflammasome is an intracellular multi-protein component composed primarily of nucleotide-binding oligomerization domain-like receptor (NLR) family and the pyrin and hematopoietic interferon-inducible nuclear domain protein family [20]. NLR protein-3 (NLRP3) is an important member of NLR family that recognizes and is activated by pathogen-associated molecular patterns or damage-associated molecular patterns [21, 22].

Recent reports have linked PM_2.5_ or smoking-induced pyroptosis to the development of COPD. In an in vitro COPD model exposed to cigarette smoke extract (CSE), NLRP3 activity was upregulated and augmented upon COPD exacerbation [23]. CSE induced pyroptosis in human bronchial epithelial cells via the NLRP3/caspase-1 pathway [24]. PM_2.5_ induced lung inflammation and pyroptosis in mice via the NLRP3/caspase-1 pathway [25–27] and exacerbated cigarette smoke-induced changes in COPD animal model [28, 29]. However, the mechanism underlying the inflammation and cell death in the lungs of patients with COPD remains unclear. Therefore, we evaluated the effects of combined exposure to cigarette smoking and PM_2.5_ in real-world patients with COPD, smoking-exposed in vivo mouse model, and human cellular in vitro model.

## Methods

### Ambient PM_2.5_ concentration and clinical parameters in patients with COPD

We conducted a prospective cohort study at four referral hospitals in South Korea between 2019 and 2020, to assess the association between ambient PM_2.5_ concentrations and clinical parameters in patients with COPD over 1-year period. The detailed protocols [30, 31] and preliminary results [32] have been previously published. The study was individually approved by the Institutional Review Boards. All participants provided written informed consent.

Here, we performed a post-hoc analysis on correlations between PM_2.5_ exposure level and clinical parameters according to patients’ smoking status. The PM_2.5_ exposure level was measured using the actual exposure concentration of PM_2.5_. Clinical parameters included the Saint George’s respiratory questionnaire specific for COPD (SGRQ-C) and the number of acute exacerbations.

### Animal model

All animal care and experimental procedures were approved by Institutional Animal Care and Use Committee of Asan Medical Center (approval number 2020-12-342). Female C57BL/6 mice (*n* = 22), 7 weeks old, weighing 18–19 g, were obtained from Orient Bio (Seongnam, Republic of Korea). Mice were randomly classified into four groups: control, smoking exposure, PM_2.5_ exposure, and smoking and PM_2.5_ combination exposure. The PM_2.5_ exposure group was exposed to 50 µg of PM_2.5_ per mouse (50 µg of PM_2.5_ in 30 µl of phosphate-buffered saline [PBS]) by intratracheal instillation every 3 days for 4 weeks (10 times in total) [33]. The PM_2.5_ exposure dose was based on preliminary experiments (Fig. S1). The control group was administered 30 µl of PBS per mouse by intratracheal instillation every 3 days for 4 weeks (10 times in total). The smoking exposure group was exposed to cigarette smoke for 5 weeks (5 days/week) using 12 commercial cigarettes per day (4 cigarettes/session, 3 sessions/day, 8.0 mg tar/cigarette, and 0.70 mg nicotine/cigarette, Camel, R. J. Reynolds Tobacco Company, Winston-Salem, NC, USA) via a whole-body apparatus according to a previously described protocol [34–39]. The control group inhaled only clean-room air (filtered air). In the real-world, people are exposed to PM, and to cigarette smoke superimposed to PM when smoking. Thus, experimental mice were exposed to PM_2.5_ prior to cigarette smoke. All mice were anesthetized by inhaled isoflurane and euthanized 6 weeks after the abovementioned treatment, and lung tissues and bronchoalveolar lavage fluid (BALF) were collected for further experiments.

### Preparation of PM_2.5_ and CSE

PM_2.5_ was obtained by separating PM_10_ certified reference material (i.e., ERM-CZ100) using a modified sedimentation method [40]. First, 500 mg of ERM-CZ100 (Sigma-Aldrich, St. Louis, MO, USA) was dispersed in 100 mL of 99.9% anhydrous ethanol (Samchun Chemicals, Seoul, Republic of Korea) and sonicated (DH.WUC. A03H, Daihan Scientific, Daegu, Republic of Korea) for 15 min. The sonicated PM_10_ solution was sedimented at room temperature for 30 min. To separate PM_2.5_ and solvent, 50 mL of the supernatant was centrifuged at 3220 x *g* for 5 min (Centrifuge 5810, Eppendorf, Hamburg, Germany). After removing the supernatant, the PM_2.5_ was collected in a glass vial and dried at 80 °C in a vacuum oven to remove residual ethanol.

CSE was prepared as described previously [41]. Briefly, the smoke from 4 cigarettes (Camel, R. J. Reynolds Tobacco Company) was drawn into a glass syringe containing 50 ml serum-free RPMI 1640 medium (Welgene, Gyeongsan, Republic of Korea). The preparation was deemed 100% CSE. A 3% working solution was prepared by diluting the stock with culture medium and the remaining stock was stored at -80 ℃.

### Analysis of inflammatory cells in BALF

The trachea was catheterized and perfused with 2 ml PBS. The liquid and cellular fractions of BALF were separated by centrifugation at 439 x *g* (2200 rpm) for 10 min at 4 ℃ (Combi R515 centrifuge, Hanil Science, Daejeon, Republic of Korea). The cell pellet was suspended in PBS, seeded onto a slide, and stained with Diff-Quick (Sysmex, Kobe, Japan). The number of inflammatory cells in BALF on each slide was counted under a light microscope.

### Histological analysis

After ligating the right main bronchus, the left lung was inflated at a constant pressure of 15 cm H_2_O with 0.5% low melting-point agarose (Invitrogen, Carlsbad, CA, USA). The left lung tissue was sectioned and fixed in 10% formalin for further histological examination. Section (5 μm) of paraffin-embedded lung tissue were prepared and stained with hematoxylin and eosin (H&E). Emphysematous changes were assessed by measuring the mean linear intercept (MLI) [42], which is a measurement of the mean interalveolar septal wall distance determined by the number of interruptions in 1 mm alveolar wall lines. Four lines were drawn in each field, and each mouse was examined in at least five random fields.

### TUNEL assay

End labeling of exposed 3′-OH ends of DNA fragments in paraffin-embedded lung tissue was performed using the terminal deoxynucleotidyl transferase dUTP nick end labeling (TUNEL) DeadEnd Colorimetric kit (Promega, Madison, WI, USA). Cell nuclei were counterstained with Methyl Green (Vector Laboratories, Burlingame, CA, USA). Slides were mounted with mounting medium (HIGHDEF IHC fluoromount, Enzo Life Sciences, Farmingdale, NY, USA) and examined under a light microscope at 400X magnification. The number of TUNEL-positive cells was assessed in 10 random fields per mouse. Among the total nuclei, the percentage of TUNEL-positive cells was counted for each image, and a mean was calculated for each mouse.

### Cell culture and treatment

Human bronchial epithelial cells (BEAS-2B, #CRL-9609) were obtained from the American Type Culture Collection and were cultured in RPMI 1640 (Welgene) containing 10% (v/v) fetal bovine serum (Gibco, Thermo Fisher Scientific, Waltham, MA, USA), 100 µg/ml streptomycin, and 100 U/ml penicillin in a humidified atmosphere with 5% CO_2_. Confluent cells were detached using 0.25% trypsin and 0.05% ethylenediaminetetraacetic acid (Gibco, Thermo Fisher Scientific) for 3 min, and aliquots were subcultured. BEAS-2B cells were treated with concentrations of 20 µg/ml and 3% for PM_2.5_ and CSE, respectively, for 24 h to assess the effects of the caspase-1 inhibitor VX765 (20 µM; S2228, Selleckchem, Houston, TX, USA) and the siRNA (40 nM; si-NLRP3, 5′-CAUCAUUCCCGCUAUCUUUTT − 3′ and 60 nM; si-Caspase-1, 5′-GAG GAA AUU UUC CGC AAG G -3′,Bioneer, Daejeon, Republic of Korea). BEAS-2B cells were transfected with si-NLRP3 or si-Caspase-1 using the Lipofectamine RNAiMAX reagent (Invitrogen, Waltham, MA, USA). Transfection efficiency was determined by quantitative real-time polymerase chain reaction (qRT-PCR) and Western blot. The working concentrations of PM_2.5_ and CSE solutions were based on preliminary experiments (Fig. S2). Beas-2B cells were exposed to various concentrations of PM_2.5_ (5, 25, and 100 µg/ml) and CSE (0.5, 1, 3, and 5%) solutions. Subsequently, the cells were treated with combined PM_2.5_ and CSE solutions, including 3% CSE, 3% CSE + PM_2.5_ 10 µg/ml, 3% CSE + PM_2.5_ 20 µg/ml, 5% CSE, 5% CSE + PM_2.5_ 20 µg/ml, PM_2.5_ 10 µg/ml, and PM_2.5_ 20 µg/ml. Notably, cells treated with 3% CSE + PM_2.5_ 20 µg/ml exhibited marked cytotoxicity compared with those treated with 3% CSE alone, thus establishing it as the selected working concentration.

### Cell viability assay

Cell viability was assessed using the 3-(4,5-dimethylthiazol-2-yl)-2,5-diphenyltetrazolium bromide (MTT) assay. BEAS-2B cells were seeded in a 96-well culture plate at a density of 5 × 10^3^/well and then treated with PM_2.5_ and CSE for 24 h. 20 µl of MTT (at a concentration of 5 mg/ml, Sigma-Aldrich) was added, and the cells were incubated for 2 h in a humidified incubator. 100 µl of dimethyl sulfoxide (DMSO) was added after discarding the supernatant to dissolve the formazan, and the optical density at 570 nm (OD570nm) was measured. The viability of the untreated cells (control) was defined as 100%, and the cell viability of all other groups was calculated separately from that of the control group.

### Lactate dehydrogenase (LDH) release assay and cytokine measurement

An LDH cytotoxicity assay kit (EZ-CYTOX, DoGenBio, Seoul, Republic of Korea) was used to examine the LDH release level, and the optical density of samples was measured at 490 nm. Interferon-γ (IFN-γ) and interleukin-6 (IL-6) levels in BALF and IL-6 and IL-8 levels in cell supernatant were measured using enzyme-linked immunosorbent assay (ELISA)-based kits (R&D Systems, Minneapolis, MN, USA).

### Western blot analysis

BEAS-2B cells were treated under different conditions. The total protein was extracted from the cells using RIPA buffer (Abcam, Cambridge, UK) containing protease and phosphatase inhibitors (1 µg/ml Aprotinin, 1 µg/ml Leupeptin, 200 µM phenylmethylsulfonyl fluoride (PMSF), 1 mM Na_3_VO_4_). Then, the total protein was separated and transferred to membranes. The membranes were incubated with 5% non-fat milk and specific antibodies against NLRP3 (1:1000; ab263899, Abcam), caspase-1 (1:1000; ab207802, Abcam), and glyceraldehyde 3-phosphate dehydrogenase (GAPDH) (1:1000; 2118, Cell Signaling Technology, Beverly, MA, USA). Finally, the proteins were visualized using ImageQuant LAS 4000 (GE Healthcare, Uppsala, Sweden) or X-ray film in a darkroom.

### qRT-PCR

Trizol (Thermo Fisher Scientific) was used to extract total RNA from lung tissue and BEAS-2B cells. Revert Aid First Strand cDNA Synthesis kit (Thermo Fisher Scientific) was used to synthesize cDNA from 1 µg of total RNA. The amount of mRNA was quantified using Advanced Universal SYBR Green Supermix kit and the CFX Connect real-time PCR system (Biorad, Hercules, California, USA). All experiments were performed in duplicate. The following primers were used in lung tissues: tumor necrosis factor-α (TNF-α), IL-1α, IL-1β, IL-6, IL-18, IL-33, IFN-γ, and 18 S. The following primers were used in BEAS-2B cells: IL-6, IL-8, NLRP3, caspase-1, IL-1β, IL-18, caspase-3, caspase-7, HMGB1, caspase-8, RIPK3, and GAPDH. Primer sequences are listed in Supplementary Table. 1. The expression levels of the target genes were normalized to 18 S or GAPDH as an endogenous control gene. The relative changes were calculated using the Eq. 2^−ΔΔCt^.

### Statistical analysis

Clinical data are presented as mean ± standard deviation or number (%). The relationships between PM_2.5_ concentration level and clinical parameters were evaluated using linear regression in R Statistical Software (version 4.0.3; R Foundation for Statistical Computing, Vienna, Austria). For all variables, the experimental results are expressed as mean  ±  standard deviation. Differences between groups were compared using one-way ANOVA, followed by post hoc Tukey’s test for multiple comparisons, and Student’s t-test for two groups using GraphPad Prism software (Version 5.01, GraphPad, San Diego, CA, USA). Statistical significance was accepted for p-value < 0.05.

## Results

### Ambient PM_2.5_ deteriorated quality of life (QOL) in currently smoking patients with COPD

We evaluated the relationship between PM_2.5_ exposure levels and clinical parameters to determine the effects of cigarette smoking and PM_2.5_ exposure in patients with COPD. A total of 105 patients were included in the study with 23 (21.9%) of them being current smokers. Their mean age was 68.2 ± 7.2 years, and 97 (92.4%) were male. The mean post-bronchodilator forced expiratory volume in 1 s was 1.6 ± 0.6 L, representing 53.9 ± 16.5% of the predicted value. Mean baseline SGRQ-C score was 37.5 ± 21.8, and 41 patients (39.0%) had a history of acute exacerbation. The mean outdoor and indoor PM_2.5_ concentration were 17.2 ± 5.0 µg/m^3^ and 16.2 ± 8.4 µg/m^3^, respectively (Table 1).

**Table 1:** Baseline characteristics of the study subjects

	Total (*n* = 105)
Age, years	68.2 ± 7.2
Male sex	97 (92.4)
Current smoker	23 (21.9)
Post-bronchodilator FEV1, L	1.6 ± 0.6
Post-bronchodilator FEV1, % predicted	53.9 ± 16.5
SGRQ-C score	37.5 ± 21.8
History of acute exacerbation	41 (39.0)
Outdoor PM_2.5_ concentration, µg/m^3^	17.2 ± 5.0
Indoor PM_2.5_ concentration, µg/m^3^	16.2 ± 8.4

Data are presented as the mean ± standard deviation or number (%), unless otherwise indicated. FEV1, forced expiratory volume in 1 s; SGRQ-C, Saint George’s respiratory questionnaire specific for COPD; PM_2.5_, particulate matter of diameter ≤ 2.5 μm

**Fig. 1:**
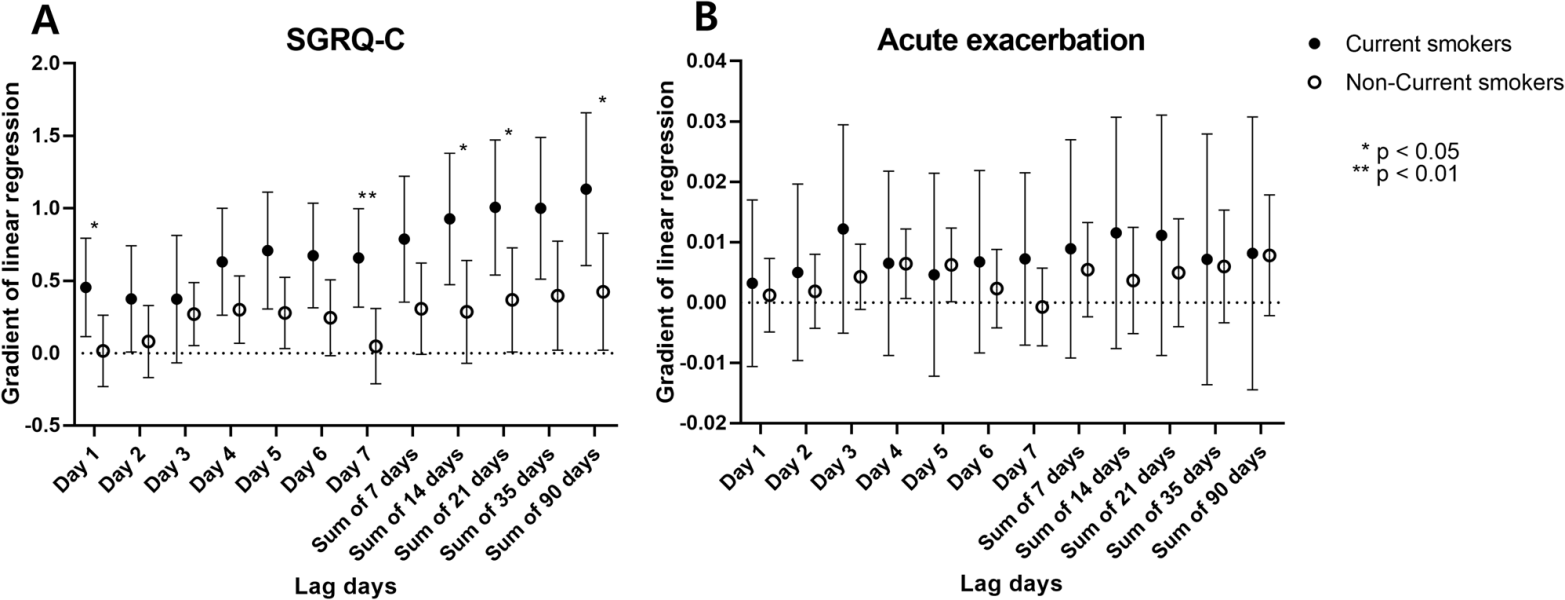
Correlation between clinical outcomes and actual PM_2.5_ exposure levels in the days preceding the evaluation. (**A**) SGRQ-C scores. (**B**) Number of acute exacerbations. The significance is determined by linear regression. * *P* < 0.05, ** *P* < 0.01, *** *P* < 0.001. SGRQ-C, Saint George’s Respiratory Questionnaire for COPD

The SGRQ-C score significantly correlated with the PM_2.5_ exposure level in the days preceding the assessment in currently smoking patients. However, there was no correlation between SGRQ-C score and PM_2.5_ exposure level in non-smoking patients (Fig. 1A). Regardless of smoking status, there was no significant correlation between the number of acute exacerbations and the PM_2.5_ exposure level (Fig. 1B), although a slightly higher correlation was noted in current smokers. These results suggest that exposure to ambient PM_2.5_ deteriorates QOL and may worsen prognosis in currently smoking patients with COPD. To investigate the underlying mechanisms, we performed subsequent in vivo and in vitro experiments.

### Smoking and PM_2.5_ exposure induced lung injury and increased inflammation in vivo

**Fig. 2:**
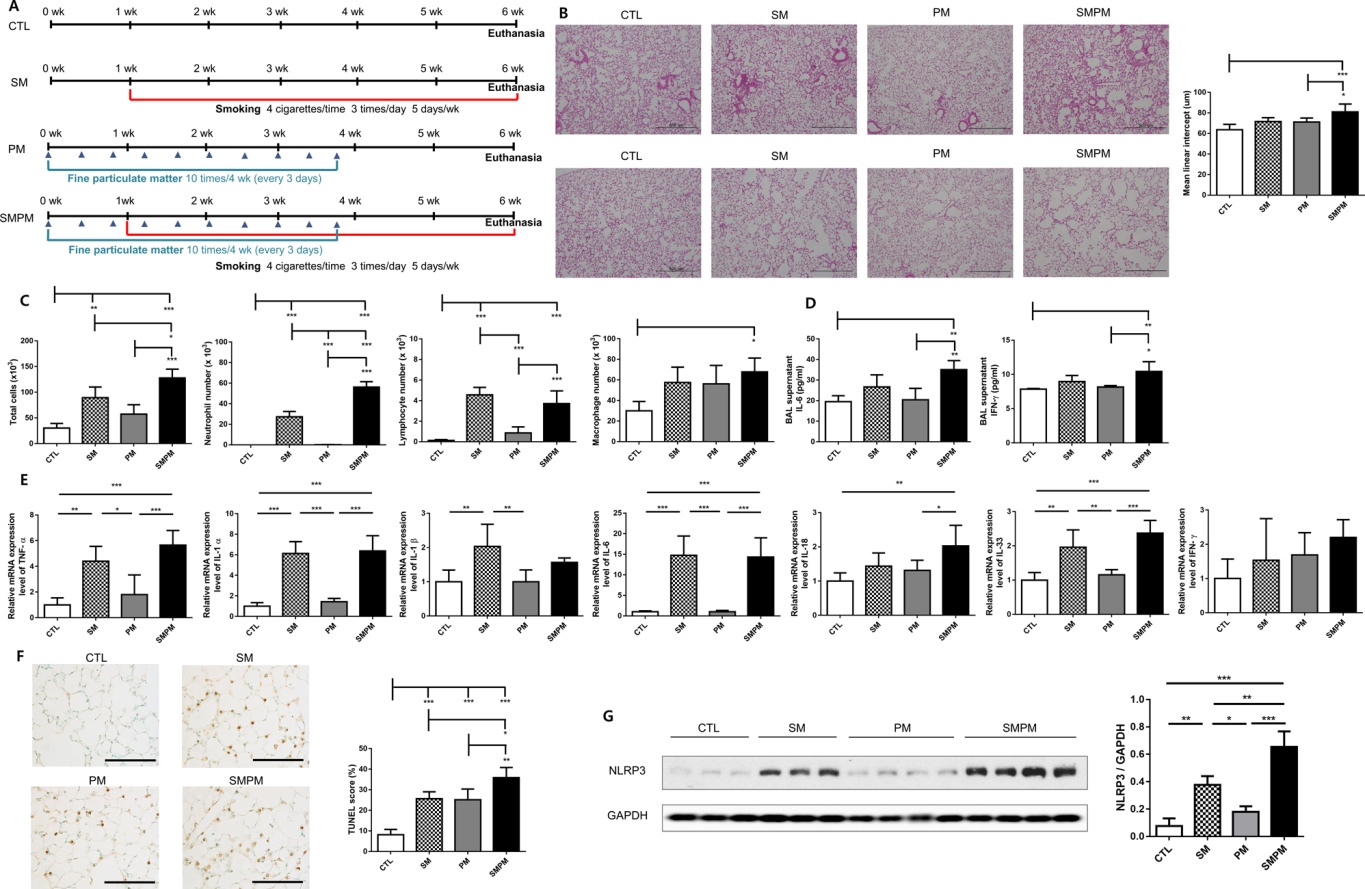
Smoking and PM_2.5_ triggered lung injury and increased inflammation. (**A**) Timeline of COPD model and PM_2.5_ treatment. (**B**) Representative H&E stained lung tissue from mice in control, smoking, PM_2.5_, and smoking with PM_2.5_ groups (100X original magnification, scale bar = 500 μm). The upper images show peribronchial inflammatory cell infiltration. The lower images show alveolar destruction. The MLI of lung tissue samples from each group (*n* = 4 control mice, *n* = 4 SM mice, *n* = 5 PM mice, and *n* = 8 SMPM mice). (**C**) Total number of cells in the BALF infiltrating the airways. Differential cell numbers of BALF in each group (*n* = 4 control mice, *n* = 4 SM mice, *n* = 6 PM mice, and *n* = 4 SMPM mice). (**D**) Levels of cytokines IL-6 and IFN-γ in BALF determined by ELISA (*n* = 4 control mice, *n* = 4 SM mice, *n* = 4 PM mice, and *n* = 5 SMPM mice). (**E**) The relative mRNA levels of TNF-α, IL-1α, IL-1β, IL-6, IL-18, IL-33, and IFN-γ in lung tissues (*n* = 4 control mice, *n* = 4 SM mice, *n* = 6 PM mice, and *n* = 5 SMPM mice). (**F**) Representative TUNEL images of lung tissue of mice and TUNEL score (%) of each group (*n* = 4 control mice, *n* = 4 SM mice, *n* = 5 PM mice, and *n* = 6 SMPM mice, 400X original magnification, scale bar = 100 μm). (**G**) NLRP3 expression levels in lung tissue of mice (*n* = 3 control mice, *n* = 3 SM mice, *n* = 4 PM mice, and *n* = 4 SMPM mice. The data are represented as mean ± SD. The significance is determined using one-way ANOVA, followed by post hoc Tukey’s test. * *P* < 0.05, ** *P* < 0.01, *** *P* < 0.001. H&E, Hematoxylin and eosin; MLI, Mean linear intercept; SD, standard deviation; CTL, control; SM, smoking; PM, PM_2.5_; SMPM, smoking and PM_2.5_

To investigate the histological changes induced by cigarette smoking and PM_2.5_ separately or together, experimental mice were classified into four groups: control, smoking, PM_2.5_, and smoking and PM_2.5_ combination (Fig. 2A). H&E-stained lung tissue slides from each group revealed that combined exposure to cigarette smoke and PM_2.5_ resulted in greater peribronchial infiltration of inflammatory cells and greater alveolar destruction compared with those observed in separate exposure to cigarette smoke or PM_2.5_ (Fig. 2B). The lymphocytes and neutrophils infiltration was reduced in the BALF of PM_2.5_ exposed mice compared with that of the smoking exposed mice; however, the combined exposure to smoking and PM_2.5_ resulted in greater infiltration of these cells (Fig. 2C).

The levels of cytokines, such as IL-6 and IFN-γ, were significantly increased in the BALF of mice exposed to a combination of smoking and PM_2.5_ compared with those of the control (Fig. 2D) [43, 44]. Moreover, the mRNA expression of proinflammatory cytokines, such as TNF-α, IL-1α, IL-6, IL-18, and IL-33, was significantly increased in the smoking and PM_2.5_ combination group compared with those in the control group (Fig. 2E).

Next, we determined whether the combination of cigarette smoke and PM_2.5_ would affect cell death response compared with exposure to cigarette smoke or PM_2.5_ separately. Cell apoptosis was most prominent in the smoking and PM_2.5_ combination group (Fig. 2F). To evaluate other types of cell death, such as pyroptosis, we assessed NLRP3 expression levels using western blotting. NLRP3 expression was significantly upregulated in the smoking group compared with that in the control, but not upregulated in the PM_2.5_ group; moreover, it peaked in the smoking and PM_2.5_ combination group (Fig. 2G).

### PM_2.5_ aggravated CSE-induced inflammation in BEAS-2B cells

**Fig. 3:**
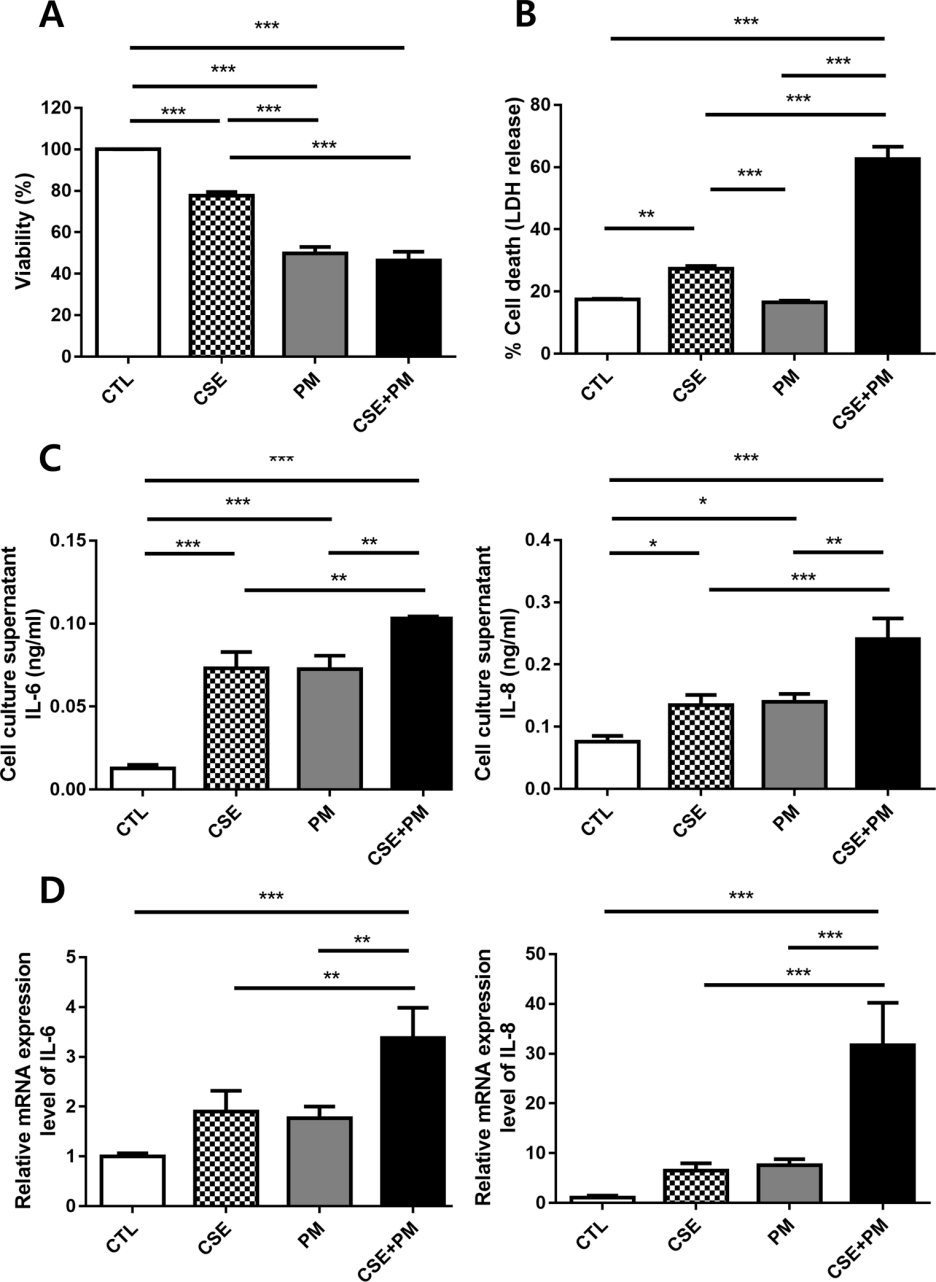
PM_2.5_ aggravated cigarette smoke extract-induced inflammation in BEAS-2B cells. (**A**) PM_2.5_ and cigarette smoke extract decreased cell viability. (**B**) PM_2.5_ and cigarette smoke extract increased LDH release. (**C**) PM_2.5_ and cigarette smoke extract enhanced the release of IL-6 and IL-8 in the cell supernatant. (**D**) Relative mRNA expression level of IL-6 and IL-8. The data are represented as mean ± SD (*n* = 3). The significance is determined using one-way ANOVA, followed by post hoc Tukey’s test. * *P* < 0.05, ** *P* < 0.01, *** *P* < 0.001. CTL, control; CSE, cigarette smoke extract; PM, PM_2.5_; CSE + PM, cigarette smoke extract and PM_2.5_

BEAS-2B cell viability was assessed to determine whether CSE and PM_2.5_ induce inflammatory reaction and cell death in cell lines. The MTT assay showed that CSE or PM_2.5_ alone inhibited the proliferation of BEAS-2B cells compared with that in control (Fig. 3A). Next, we determined whether the decreased cell viability was related to inflammasome-induced pyroptosis. In a subsequent experiment, pyroptotic cell death was evaluated by measuring LDH release [45]. The combination of CSE and PM_2.5_ significantly increased LDH release compared with that in the CSE or PM_2.5_ group (Fig. 3B).

The IL-6 and IL-8 levels in the cell culture supernatants were then assessed by ELISA. CSE or PM_2.5_ alone upregulated inflammation in BEAS-2B cells, whereas the combination of CSE and PM_2.5_ significantly increased both cytokine levels compared with that in CSE or PM_2.5_ alone (Fig. 3C). To confirm our findings, we measured the transcriptional levels of these cytokines at different exposure levels using qRT-PCR, and the results were consistent with those assessed by ELISA (Fig. 3D).

### PM_2.5_ and CSE exposure upregulated pyroptosis-related genes expression

**Fig. 4:**
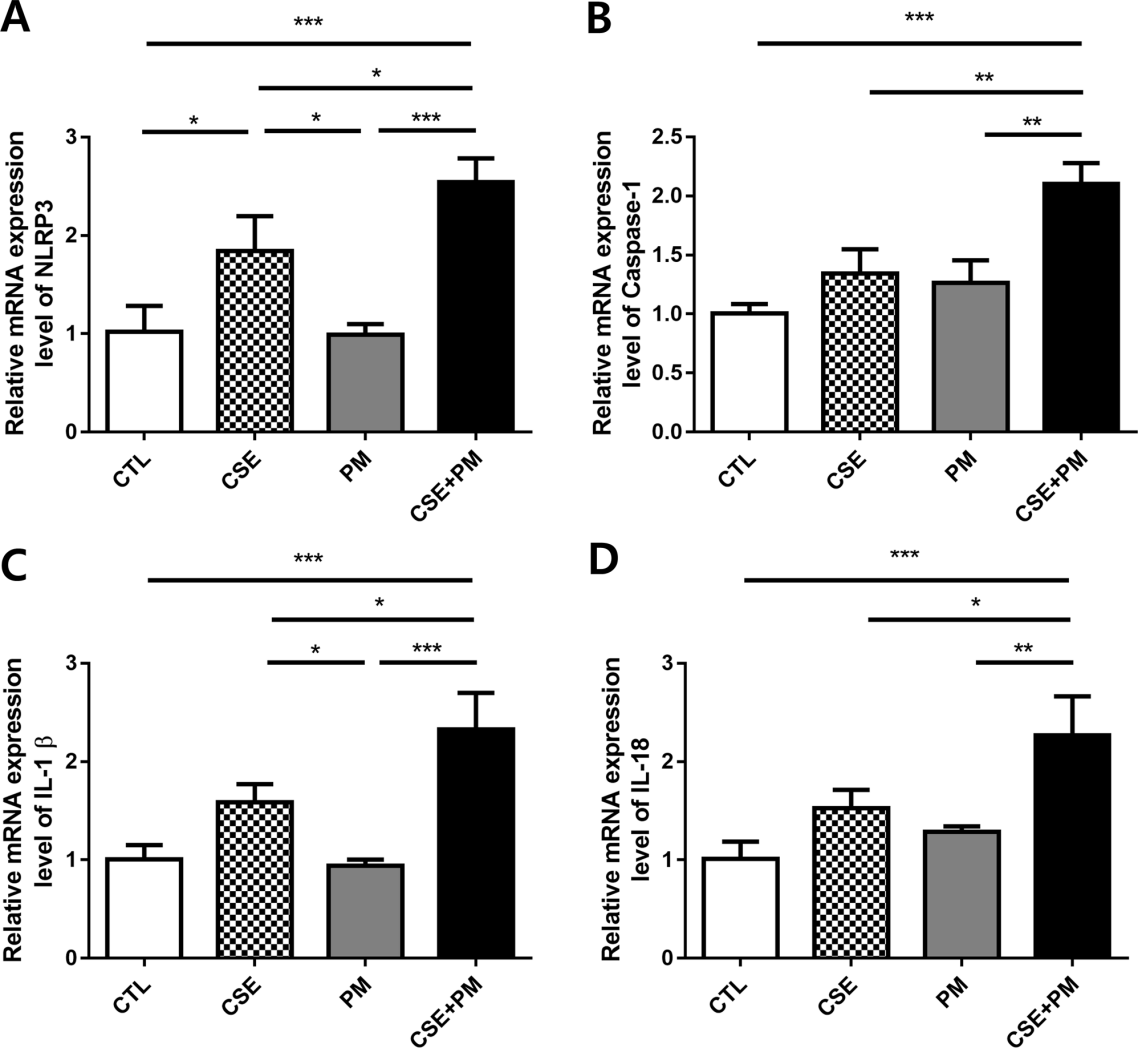
Exposure to PM_2.5_ and cigarette smoke extract upregulated expression of pyroptosis-related genes in BEAS-2B cells. (**A**) Relative mRNA expression level of NLRP3. (**B**) Relative mRNA expression level of Caspase-1. (**C**) Relative mRNA expression level of IL-1β. (**D**) Relative mRNA expression level of IL-18. The data are represented as mean ± SD (*n* = 3). The significance is determined using one-way ANOVA, followed by post hoc Tukey’s test. * *P* < 0.05, ** *P* < 0.01, *** *P* < 0.001

BEAS-2B cells were exposed to CSE and PM_2.5_ for 24 h to elucidate the mechanism underlying their inducibility to inflammation and cell death. The transcriptional levels of pyroptosis-related genes, such as NLRP3, caspase-1, IL-1β, and IL-18, were significantly upregulated in the CSE group compared with those in the control, but they were not upregulated in the PM_2.5_ group; moreover, the expression of pyroptosis-related genes peaked in the CSE and PM_2.5_ combination group (Fig. 4A-D).

We determined whether CSE and PM_2.5_ were also associated with apoptosis- or necrosis-related gene expression. When we compared the mRNA expression levels of the apoptosis-related genes such as caspase-3 and caspase-7, we found that caspase-3 expression was elevated in the CSE and CSE and PM_2.5_ combination groups compared with that in the control, whereas no difference in the expression of caspase-7 was observed (Fig. S3A). When comparing the mRNA expression levels of necrosis-related genes, such as HMGB1, caspase-8, and RIPK3, no significant difference between groups was observed (Fig. S3B).

These results demonstrated that exposure to the combination of CSE and PM_2.5_ could decrease BEAS-2B cell viability while increasing inflammation by inducing pyroptosis. Therefore, subsequent studies were conducted to determine how the combination of CSE and PM_2.5_ induces pyroptosis.

### PM_2.5_ and CSE induce pyroptosis via the NLRP3 inflammasome

**Fig. 5:**
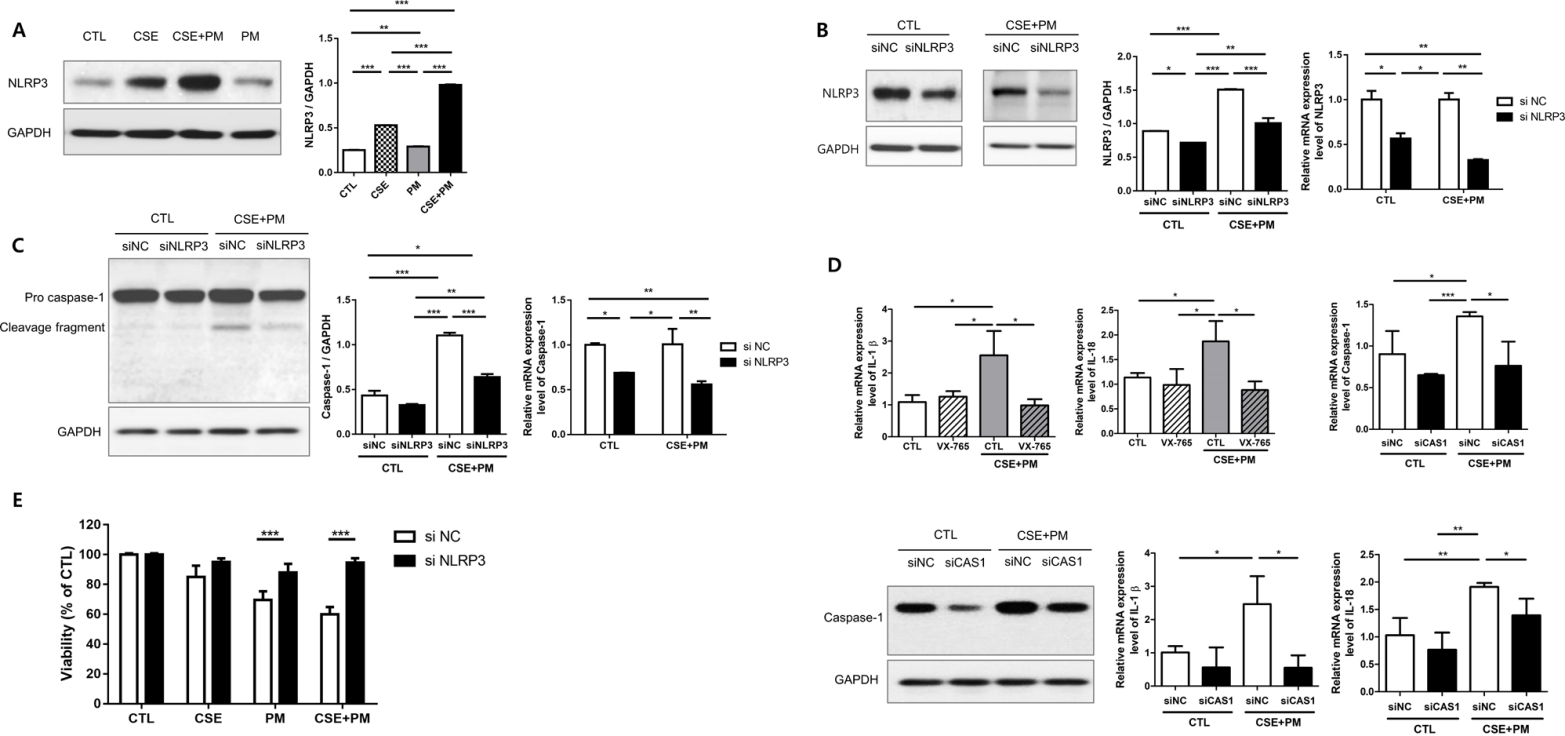
NLRP3 is necessary for CSE and PM_2.5_ induced pyroptosis. (**A**) NLRP3 expression levels in BEAS-2B cells after treatment with CSE and PM_2.5_. (**B**) Transfection with siNLRP3 decreased the protein and mRNA levels of NLRP3. (**C**) Transfection with siNLRP3 decreased the protein and mRNA levels of activated caspase-1. (**D**) Inhibition of caspase-1 decreased mRNA levels of IL-1β and IL-18. (**E**) Silencing of NLRP3 significantly increased cell viability. The data are represented as mean ± SD (*n* = 2 B and C, *n* = 3 D, *n* = 4 E) from three independent experiments. The significance is determined using one-way ANOVA, followed by post hoc Tukey’s test. As for (E), the significance is displayed only between siNC and siNLRP3 for each group. * *P* < 0.05, ** *P* < 0.01, *** *P* < 0.001. NC, negative control

We evaluated the interaction between NLRP3 and caspase-1 to determine the role of NLRP3 in the activation of caspase-1 by CSE and PM_2.5_ exposure. After treating BEAS-2B cells with CSE and PM_2.5_, western blotting was performed to assess the protein expression level of NLRP3. Exposure to the combination of CSE and PM_2.5_ increased NLRP3 expression (Fig. 5A).

BEAS-2B cells were transfected with siNLRP3 to confirm the role of NLRP3 in the combined exposure to CSE and PM_2.5_. Compared with transfection with the siRNA negative control (siNC), transfection with siNLRP3 reduced NLRP3 protein and mRNA expression, both with and without CSE and PM_2.5_ combination. NLRP3 protein and mRNA levels were significantly lower in the combination of CSE and PM_2.5_ than in the control group (Fig. 5B). Moreover, transfection with siNLRP3 decreased the relative protein and mRNA expression levels of activated caspase-1 (Fig. 5C). Furthermore, inhibition of caspase-1 decreased the release of IL-1β and IL-18 (Fig. 5D), and cell viability was also restored by siNLRP3 (Fig. 5E). These results demonstrate that exposure to CSE and PM_2.5_ induces pyroptosis via NLRP3/caspase-1 pathway.

## Discussion

In our study, high levels of ambient PM_2.5_ were associated with higher SGRQ-C scores in currently smoking patients with COPD. Combined exposure to cigarette smoke and PM_2.5_ was associated with increased inflammatory cell infiltration and inflammatory cytokine release in mouse lung. Exposure to a combination of CSE and PM_2.5_ reduced cell viability and upregulated pyroptosis-related gene transcription in BEAS-2B cells. NLRP3 silencing with siRNA reduced pyroptosis and restored cell viability. Therefore, PM_2.5_ aggravates smoking-induced airway inflammation and cell death via pyroptosis.

Cigarette smoke-induced airway epithelial damage is reported to induce airway remodeling, resulting in COPD [46, 47]. The epithelial changes in COPD are varied, including Goblet cell metaplasia, squamous metaplasia, and basal membrane thickening [48–50]. Previous studies demonstrated cigarette smoke-induced damage on bronchial or tracheal epithelial cells, including impaired epithelial barrier function, cilia toxicity, and mucus hypersecretion [51–54]. Recently, cigarette smoking-induced changes in human circulating cells were reported, including increased circulating leukocytes and neutrophils, upregulated myeloid-derived suppressor cells, and activated natural killer cells [55–57].

There is growing evidence that PM_2.5_ exposure is associated with the recruitment and activation of inflammatory cells in lung. Exposure to PM_2.5_ significantly increased inflammatory cells, including neutrophils, lymphocytes, eosinophils, and macrophages, as well as inflammatory cytokines in BALF [14, 15, 27]. Moreover, exposure to PM_2.5_ was associated with alveolar destruction, septal thickening, emphysematous changes, airway remodeling, and inflammatory cell infiltration [27, 28]. Exposure to PM_2.5_ and cigarette smoke has been reported to aggravate inflammation and structural changes in the lungs [28, 29]. In our experiment, there was a significant increase in inflammatory cells and cytokines in the BALF supernatant, as well as an increase in MLI and TUNEL-positive cells and marked emphysematous changes in the lung tissue of mice exposed to both PM_2.5_ and cigarette smoke. These findings suggest that PM_2.5_ exposure could aggravate cigarette smoking-induced airway inflammation and structural destruction in the lungs of patients with COPD. Moreover, PM exposure is also associated with altered immune cell phenotype [58, 59] and immunosuppressive lung microenvironment, which is highly populated by tolerogenic dendritic cells, macrophages, and myeloid-derived suppressor cells [60]. Further studies are needed on PM-induced non-inflammatory lung injury.

Previously, the Wnt5a–ERK pathway was proposed as a mechanism whereby PM_2.5_ could aggravate smoking-induced airway inflammation [29]. Meanwhile, our data revealed that the mRNA expression levels of IL-1β and IL-18 were elevated in the lung tissue of mice; thus, we could infer that pyroptosis may contribute to inflammation and lung injury caused by PM_2.5_ and cigarette smoking [24, 25, 27]. Pyroptosis is a kind of programmed cell death induced by inflammasomes [18, 19]. Among the inflammasomes, NLRP3 is known to be critical in recognizing pathogen-associated molecular patterns or damage-associated molecular patterns [21, 22]. Activated NLRP3 inflammasome triggers cleavage of pro-caspase-1, resulting in cleaved caspase-1. Thereafter, activated caspase-1 increases IL-1β and IL-18 and cleaves gasdermin D, leading to pyroptosis [24, 25, 61, 62]. Our findings supported the role of pyroptosis by demonstrating that the exposure to both PM_2.5_ and CSE decreased cell viability while upregulating transcription of NLRP3, caspase-1, IL-1β, and IL-18 in BEAS-2B cells. Moreover, siRNA-mediated NLRP3 gene silencing decreased both NLRP3 and caspase-1 levels while restoring cell viability. These findings suggest that both PM_2.5_ and CSE induce pyroptosis via the NLRP3/caspase-1 pathway. Furthermore, inactivation of the NLRP3 inflammasome could be a novel therapeutic target in COPD [24, 27, 63].

In our experiments, inflammatory and pyroptotic markers in in vivo and in vitro experiments were increased with cigarette smoke exposure, but not with PM_2.5_ alone. These markers were increased further with combined cigarette smoke and PM_2.5_ exposure. Therefore, our experiments suggest that cigarette smoke, compared with PM_2.5_, has a major impact on airway inflammation and pyroptotic cell death, and PM_2.5_ exposure could potentiate cigarette smoking-induced inflammatory reaction. This effects of combined exposure with smoking and PM_2.5_ also showed similar trends for the development of COPD clinically [64].

Higher ambient PM_2.5_ concentrations have recently been linked to an increased prevalence of COPD, lung function decline, and mortality [7–10]. PM_2.5_ and current cigarette smoking status have been suggested to have an additive effect on the risk of COPD [64]. Moreover, ambient PM_2.5_ exposure has been linked to severe respiratory symptoms and a decline in lung function, resulting in deteriorated QOL in patients with COPD [65, 66]. Previously, it was reported that biomass smoke exposure was associated with the airway-predominant phenotype of COPD, resulting in more air trapping, severe respiratory symptoms, and deteriorated QOL compared with smoking-related COPD of emphysema-predominant phenotype [67]. Exposure to PM_2.5_ was associated with systemic inflammation in patients with COPD [68], and systemic inflammation in COPD was associated with poor QOL [69, 70]. In pyroptosis, the plasma-membrane rapidly ruptures and proinflammatory intracellular contents are released, resulting in pathological inflammation [19]. Moreover, patients with stable COPD had significantly higher plasma IL-1β levels and upregulated expression of the IL1B, NLRP3, and CASP1 genes compared with that in healthy controls [71]. In our investigation, high ambient PM_2.5_ concentrations were linked to high SGRQ-C scores in currently smoking patients with COPD. Moreover, high SGRQ-C scores were associated with rapid lung function decline and frequent exacerbation [72–74]. We can conclude from this study that PM_2.5_ exposure aggravates smoking-induced airway inflammation and deteriorates QOL of patients with COPD, with local or systemic pyroptosis-mediated inflammation playing an important role. Furthermore, PM_2.5_ exposure may induce lung function decline and exacerbation in currently smoking patients with COPD.

There are some limitations in our study. First, PM_2.5_ exposure alone did not alter the total number or the differential proportions of cells in the BALF, nor did it induce peribronchial inflammatory cell infiltration. Second, a 1-week time interval between the last exposure to PM_2.5_ and euthanasia of the mice may allow clearance of PM_2.5_ by macrophages. Third, the synergistic effect of PM_2.5_ and cigarette smoke exposure was prominent in the protein composition of BALF, whereas this synergy was relatively less evident in qPCR levels of lung homogenate. Since both PM_2.5_ and cigarette smoke were delivered intratracheally, their impact on the alveolar space appears to be more pronounced. Additionally, the alterations observed in protein levels holds greater significance compared with those observed in mRNA. Fourth, although exposure to smoking and PM_2.5_ caused lung injury and cell death in our experiments, other cell death mechanisms, such as apoptosis, might be involved. Meanwhile, our results demonstrate a significant upregulation of pyroptosis-related genes and proteins, as well as restored cell viability with caspase-1 inhibition and NLRP3 silencing. Fifth, in clinical data, a larger sample size and longer study duration might show a more consistent difference in associations between current and ex-smokers. However, the correlation between SGRQ-C score and PM_2.5_ concentration in current smokers implies that the combined exposure to smoking and PM_2.5_ has an additive aggravating effect, and pyroptosis-induced systematic inflammation may be involved.

## Conclusions

In conclusion, the combined exposure to PM_2.5_ and cigarette smoking aggravates smoking-induced airway inflammation and cell death with pyroptosis being one of the dominant mechanisms. In patients with COPD, PM_2.5_ aggravates the QOL caused by concurrent smoking and may deteriorate lung function and induce exacerbation. COPD is a preventable disease caused by exposure to noxious particles with various synergistic effects. Identifying these addictive effects will contribute to our understanding of the pathogenesis of COPD and the development of effective treatment options.

### Electronic supplementary material

Below is the link to the electronic supplementary material.

Supplementary Material 1

## Data Availability

The datasets used and/or analysed during the current study are available from the corresponding author on reasonable request.

## Abbreviations

BALF: Bronchoalveolar lavage fluid
COPD: Chronic obstructive pulmonary disease
CSE: Cigarette smoke extract
DMSO: Dimethyl sulfoxide
ELISA: Enzyme-linked immunosorbent assay
GADPH: Glyceraldehyde 3-phosphate dehydrogenase
H&E: Hematoxylin and eosin
IFN: Interferon, IL: Interleukin
LDH: Lactate dehydrogenase
MLI: Mean linear intercept
MTT: 3-[4,5-imethylthiazol-2-yl]-2,5 diphenyl tetrazolium bromide
NLR: Nucleotide-binding oligomerization domain-like receptor
NLRP3: NLR protein-3
PBS: Phosphate-buffered saline
PM_2.5_: Particulate matter of diameter ≤ 2.5 μm
PMSF: Phenylmethylsulfonyl fluoride
QOL: Quality of life
qRT-PCR: Quantitative real-time polymerase chain reaction
SGRQ-C: Saint George’s respiratory questionnaire specific for COPD
TUNEL: Terminal deoxynucleotidyl transferase dUTP nick end labeling
