# Supplementary material for: Fine particulate matter aggravates smoking induced lung injury via NLRP3/caspase-1 pathway in COPD
Source: J Inflamm (Lond). 2024 Apr 24;21:13. doi: 10.1186/s12950-024-00384-z (PMC11041029; doi:10.1186/s12950-024-00384-z)
Supplement: Supplementary file 1 — Supplementary Material 1 [file 12950_2024_384_MOESM1_ESM.docx]

**Supplementary Table 1**. Quantitative PCR primer sequences.

| Gene | Forward/reverse | Primer sequence 5′-3′ |
| --- | --- | --- |
| Lung tissue |  |  |
| TNF-α | Forward  Reverse | CTT CTG TCT ACT GAA CTT CGG G  CAG GCT TGT CAC TCG AAT TTT G |
| IL-1α | Forward  Reverse | ATG TAT GCC TAC TCG TCG GG  GTG CAC CCG ACT TTG TTC TT |
| IL-1β | Forward  Reverse | ACG GAC CCC AAA AGA TGA AG  TTC TCC ACA GCC ACA ATG AG |
| IL-6 | Forward  Reverse | CAA AGC CAG AGT CCT TCA GAG  GTC CTT AGC CAC TCC TTC TG |
| IL-18 | Forward  Reverse | GCC TCA AAC CTT CCA AAT CAC  GTT GTC TGA TTC CAG GTC TCC |
| IL-33 | Forward  Reverse | ACT TCT CTG CCT ATC CAC GG  ATT GAC TTG CAG GAC AGG GA |
| IFN-γ | Forward  Reverse | CCT AGC TCT GAG ACA ATG AAC G  TTC CAC ATC TAT GCC ACT TGA G |
| 18s | Forward  Reverse | CAG CGT GGT CAG GAT AGA AC  CTT GAT TTG AAT GCA GCG GAC |
| BEAS-2B cell |  |  |
| IL-6 | Forward  Reverse | CCA CTC ACC TCT TCA GAA CG  CAT CTT TGG AAG GTT CAG GTT G |
| IL-8 | Forward  Reverse | ATA CTC CAA ACC TTT CCA CCC  TCT GCA CCC AGT TTT CCT TG |
| NLRP3 | Forward  Reverse | CTC ACG CAC CTT TAC CTG C  AAG TGT GGA AAG ATC CCA GC |
| caspase-1 | Forward  Reverse | ATG CCT GTT CCT GTG ATG TG  CAT CTG CGC TCT ACC ATC TG |
| IL-1β | Forward  Reverse | ATG CAC CTG TAC GAT CAC TG  ACA AAG GAC ATG GAG AAC ACC |
| IL-18 | Forward  Reverse | ACC AAG GAA ATC GGC CTC TA  ACC TCT AGG CTG GCT ATC TT |
| caspase-3 | Forward  Reverse | AGC AAA CCT CAG GGA AAC AT  CAT GGC TCA GAA GCA CAC AA |
| caspase-7 | Forward  Reverse | GGA CCG AGT GCC TAC ATA TC  TCT TTG TCT GTT CCG TTT CG |
| HMGB1 | Forward  Reverse | TTG TGC AAA CTT GTC GGG AG  AGA CAT GGT CTT CCA CCT CT |
| caspase-8 | Forward  Reverse | GAT GAC ATG AAC CTG CTG GA  TGT TGA TTT GGG CAC AGA CT |
| RIPK3 | Forward  Reverse | TTT ACC TGC ACG ACC AGA AC  CCT CCC TGA AAT GTG GAC AG |
| GAPDH | Forward  Reverse | AGG GCT GCT TTT AAC TCT GGT  CCC CAC TTG ATT TTG GAG GGA |

PCR conditions were as follows: 50 °C for 2 min, 95 °C for 10 min; 40 cycles of 95 °C for 15 s, and 60 °C for 1 min, for each primer.

TNF-α, tumor necrosis factor-α; IL, interleukin; IFN-γ, interferon-γ; NLRP3, nucleotide-binding oligomerization domain-like receptor protein 3; HMGB1, High mobility group box 1 protein; RIPK3, Receptor-interacting serine/threonine-protein kinase 3; GAPDH, Glyceraldehyde 3-phosphate dehydrogenase.

**Supplementary Figure 1.** Total number and differential proportions of cells in the bronchoalveolar lavage fluid of smoking and particulate matter exposed mice (n = 4 for each group). (A) Total number of cells in the bronchoalveolar lavage fluid. (B) Differential proportions of cells in the bronchoalveolar lavage fluid. The data are represented as mean ± SD. The significance is determined using Student’s *t*-test. * P < 0.05, ** P < 0.01, *** P < 0.001. SD, standard deviation; CTL, control; SM, smoking; PM, PM_2.5_; PM+SM, PM_2.5_ and smoking.

(A)


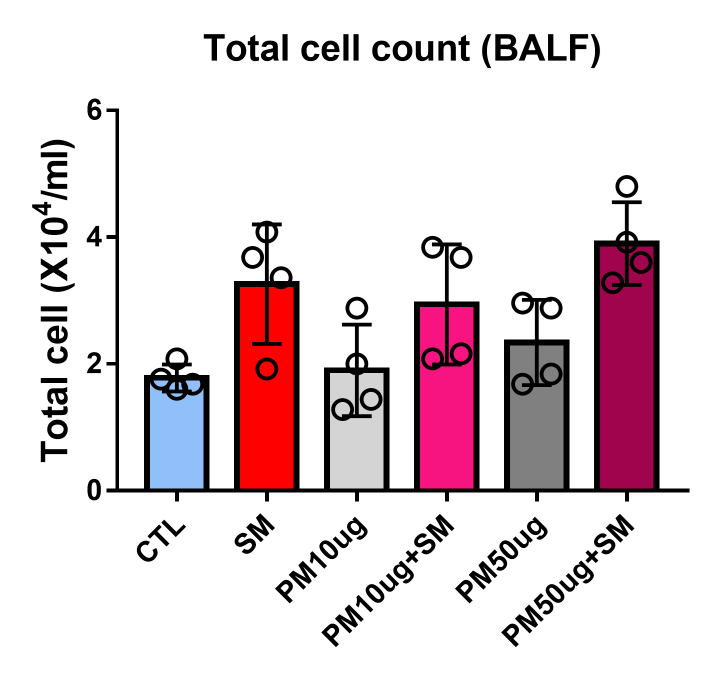


(B)


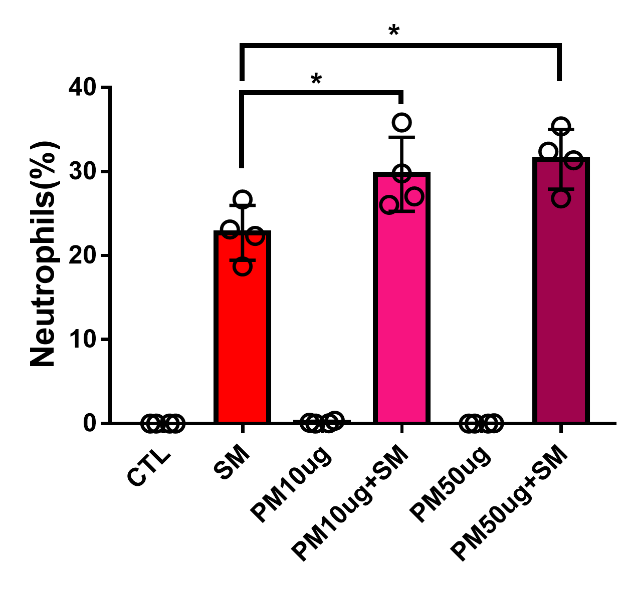

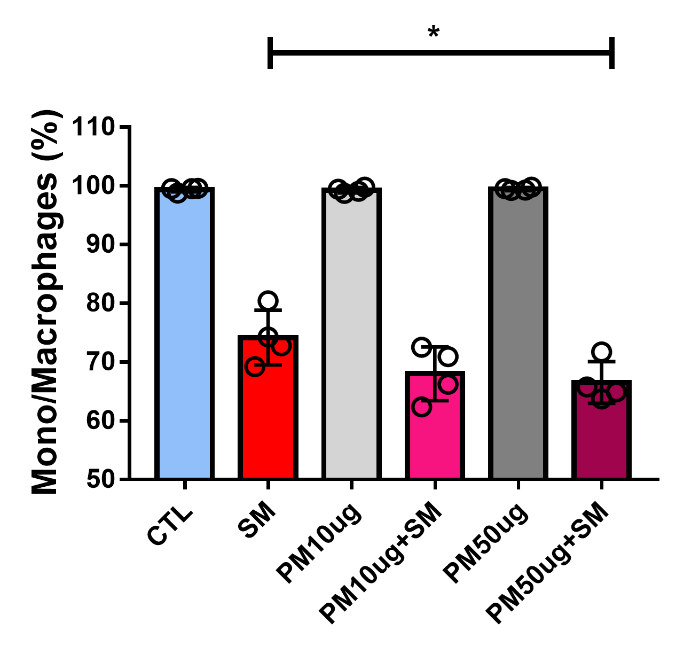


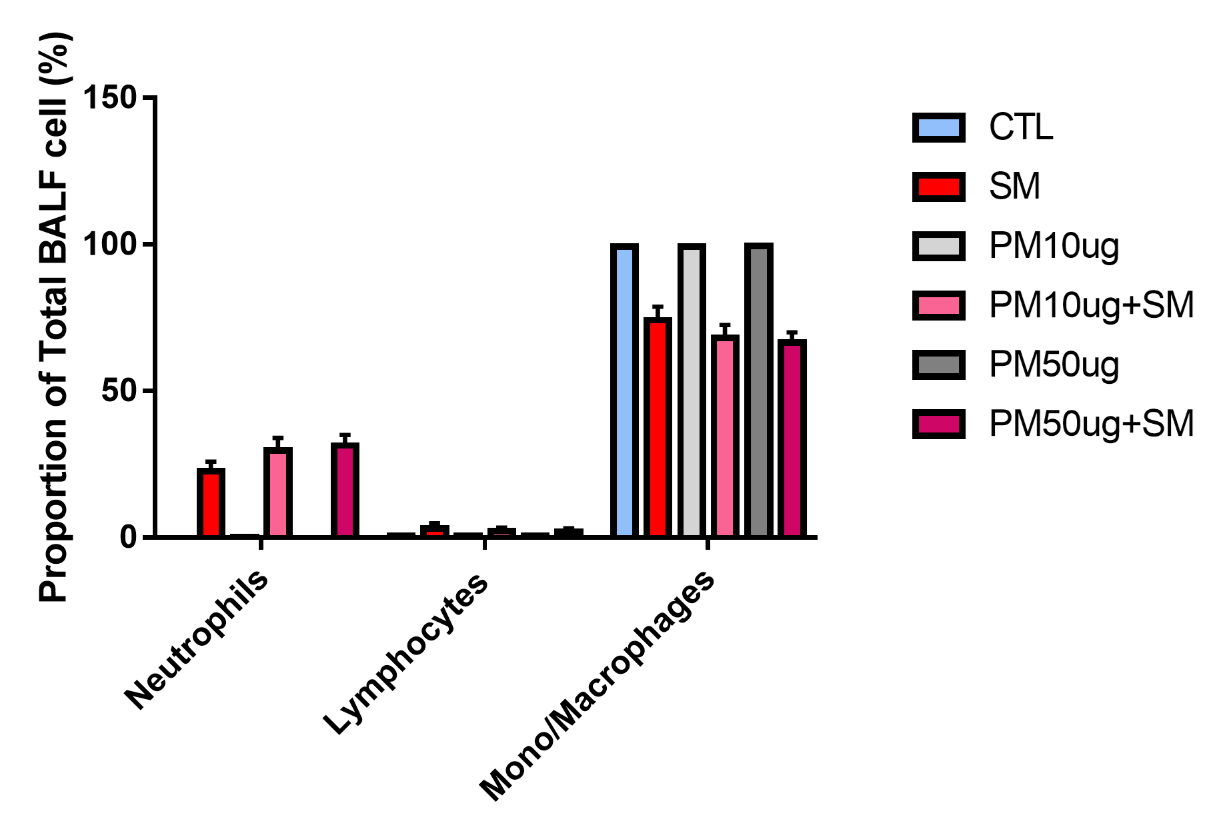


**Supplementary Figure 2.** Cytotoxicity in PM_2.5_ and cigarette smoke extract exposed BEAS-2B cells. (A) Beas-2B cells treated with PM_2.5_ and cigarette smoke extract showed cytotoxicity. (B) Beas-2B cells treated with PM_2.5_ and cigarette smoke extract combinations showed marked cytotoxicity. The data are represented as mean ± SD (n = 3). The significance is determined using Student’s *t*-test. * P < 0.05, ** P < 0.01, *** P < 0.001, **** P < 0.0001. CTL, control; CSE, cigarette smoke extract; PM, PM_2.5_; CSE+PM, cigarette smoke extract and PM_2.5_.

(A)


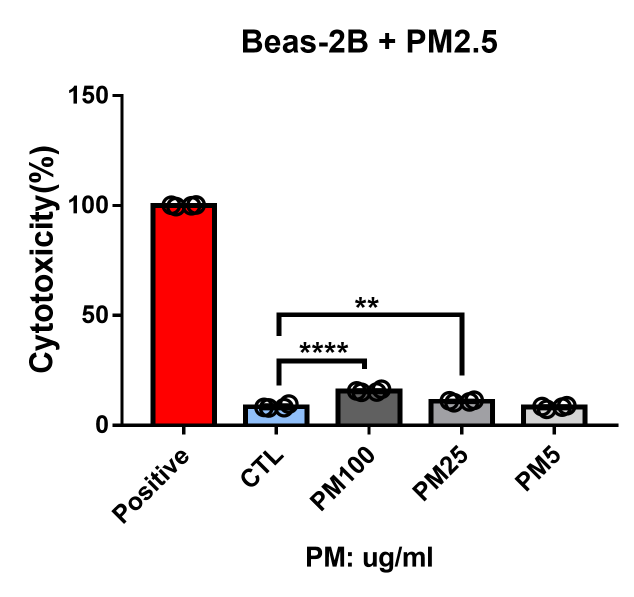

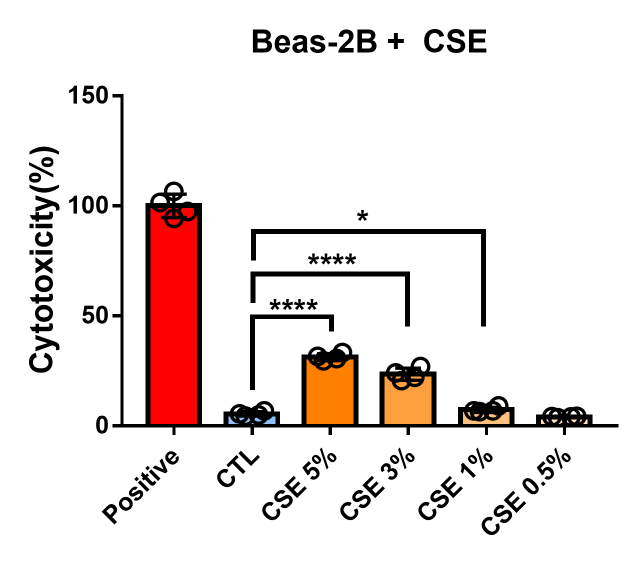


(B)


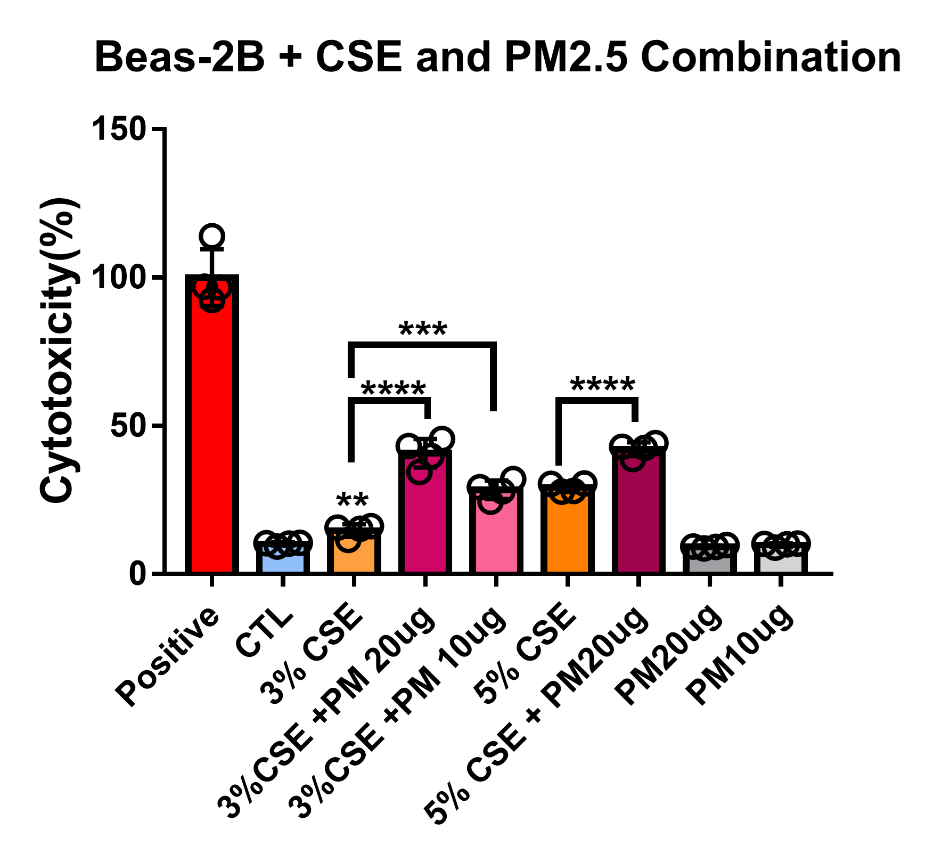


**Supplementary Figure 3.** Expression of apoptosis-related genes in BEAS-2B cells. (A) Relative mRNA expression level of Caspase-3 and Caspase-7. (B) Relative mRNA expression level of HMGB1, Caspase-8, and RIPK3. The data are represented as mean ± SD (n = 3) from three independent experiments. The significance is determined using one-way ANOVA, followed by post hoc Tukey’s test. * P < 0.05, ** P < 0.01, *** P < 0.001.

(A)


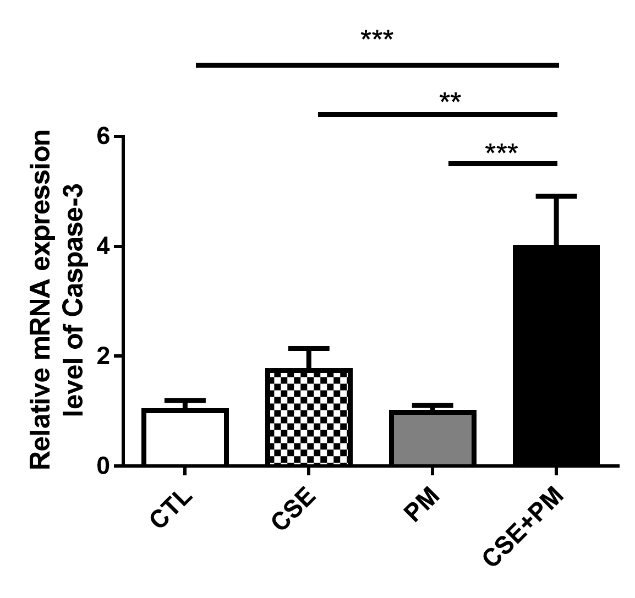
**
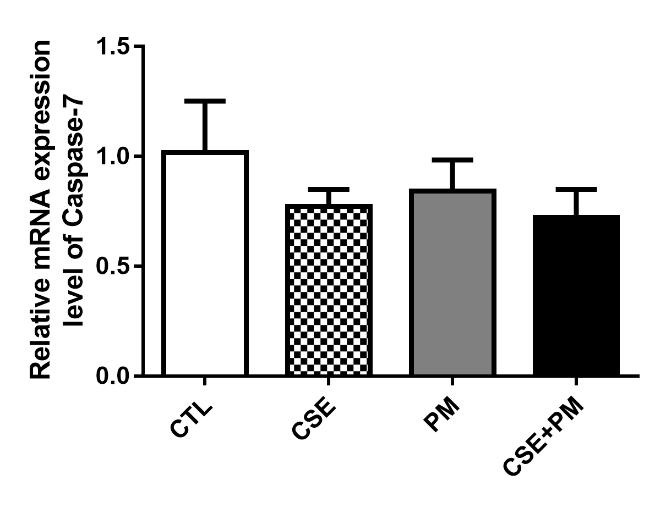
**

(B)


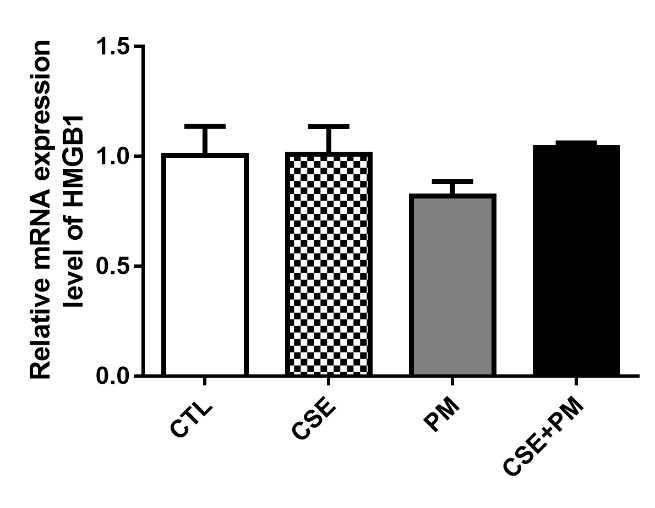

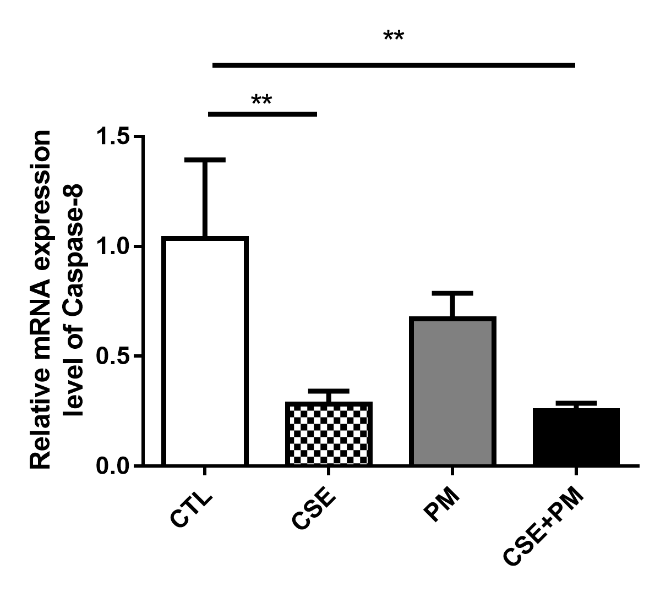


**
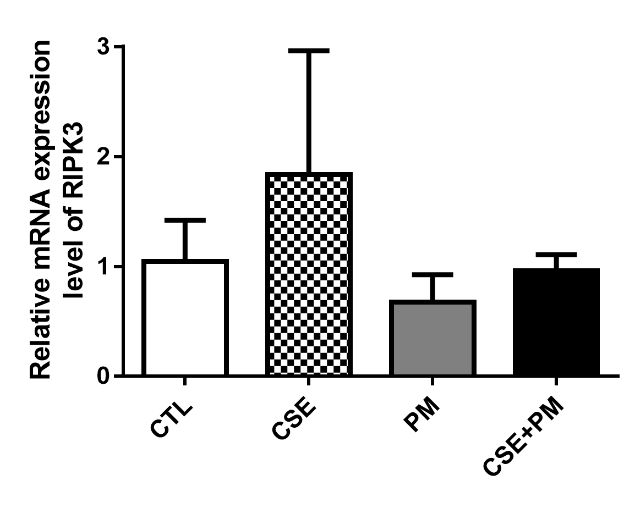
**
